# Expanding the Measurement Space of Ion Mobility-Mass Spectrometry Using Chemical Derivatizations

**DOI:** 10.1021/acsmeasuresciau.6c00210

**Published:** 2026-08-08

**Authors:** Sabrina Fernandez, Angela Acateca, Bradley B. Garrison, Heidi M. Sabatini, Christopher D. Chouinard

**Affiliations:** 2545 Clemson University, Department of Chemistry, Clemson, South Carolina 29634, United States

**Keywords:** steroids, derivatization, ion mobility-mass spectrometry, structures for lossless ion manipulations (SLIM), tandem mass spectrometry

## Abstract

Steroid analysis by mass spectrometry remains challenging because many compounds exhibit both poor ESI efficiency and subtle structural differences that limit differentiation by conventional techniques. While chemical derivatization has historically been employed to improve analytical sensitivity, its broader influence on ion mobility-mass spectrometry (IM-MS) measurements has not been systematically investigated. Here, seven hydrazine-based derivatization reagents were evaluated across a panel of 16 isomeric 11-oxosteroids using IM-MS and matrix-assisted laser desorption/ionization (MALDI)-MS. All derivatization chemistries systematically altered both molecular mass and collision cross section (CCS), producing reagent-specific shifts through CCS vs *m*/*z* measurement space. Beyond these global shifts, several reactions generated characteristic mobility fingerprints composed of multiple mobility-resolved structural populations, providing substantially richer structural information than CCS alone. The analytical utility of these measurements was demonstrated through improved differentiation of structurally similar steroid isomers using complementary descriptors including CCS, reaction stoichiometry, mobility fingerprints, and reagent-specific isotopic signatures. Finally, successful translation of the derivatization chemistry to MALDI analysis, including direct surface derivatization, established compatibility with alternative ionization methods and supports future integration with mass spectrometry imaging (MSI) workflows. Collectively, these results demonstrate that chemical derivatization fundamentally expands the analytical information accessible by IM-MS and establishes a general framework for engineering molecular measurements through differential, yet controlled, manipulation of molecular mass, CCS, and gas-phase structural diversity.

## Graphical abstract

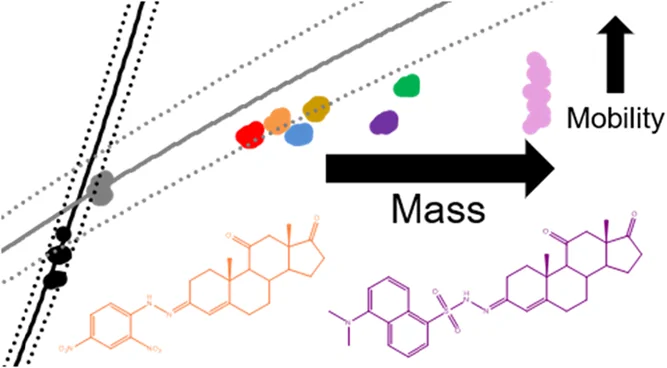

## Introduction

Steroids are a diverse class of biomolecules involved in processes ranging from development and metabolic homeostasis to sexual maturation and disease.^1–5^ Historically, immunoassays have been used to measure steroid hormones in clinical settings but suffer from both poor sensitivity and specificity.^1–5^ This has prompted a more widespread adoption of liquid chromatography-tandem mass spectrometry (LC-MS/MS) approaches to steroid quantification in a variety of biological matrixes (e.g., plasma, urine, etc.).^1–5^ Nevertheless, steroids still present numerous analytical challenges in resolution and sensitivityin sensitivity (i.e., due to their structural similarity, low/wide-ranging endogenous concentrations, and poor ionization efficiency with electrospray ionization (ESI)). Many steroids differ only in the position or stereochemistry of methyl groups, oxygen-containing functional groups (e.g., carbonyls and α/β-hydroxyls), or carbon–carbon double bonds. Furthermore, many lack a permanent charge and/or highly acidic/basic site, therefore producing weak signal with ESI.

Derivatization has been a cornerstone of the LC-MS-based steroid analysis field for many decades.^6^ Originally developed to increase volatility and thermal stability in gas chromatography–mass spectrometry (GC-MS) methods, the motivation has shifted toward improving ionization efficiency (and thus sensitivity) in LC-MS methods.^7^ A wide variety of derivatization reagents have been developed for both positive and negative ESI, as well as for other common ionization techniques such as atmospheric pressure chemical ionization (APCI). These have primarily focused on reaction with oxygen-containing functional groups, including carbonyl derivatization by Girard’s reagents and hydroxyl derivatization by acetylation.^6^

At the same time, ion mobility spectrometry (IMS) has demonstrated promise for resolution of isomeric steroids based on differentiation of molecular shape and size, as opposed to only *m*/*z*-based differentiation in MS.^8^ Generally, IMS techniques separate ions based on differences in their size, shape, and charge as they collide with neutral gas molecules while under the influence of an electric field.^9^ Coupled with MS (i.e., IM-MS), several IMS techniques have been demonstrated for steroid analysis including drift tube (DTIM),^10–20^ traveling wave (TWIM),^21–37^ and differential mobility/high field asymmetric waveform (DMS/FAIMS).^38–46^ However, the rigidity and modest structural differences between steroid isomers have challenged IMS separations, prompting development of high-resolution (resolving power, *R*_p_ ≥ 200) techniques such as trapped IMS (TIMS),^47–52^ cyclic IMS (cIMS),^53,54^ and structures for lossless ion manipulations (SLIM).^55,56^ Nevertheless, the nearly identical collision cross sections (CCS) of many steroid isomers such as testosterone and epitestosterone preclude their resolution as native compounds even with the highest performance IM-MS instruments; even ultrahigh-resolution long-pass approaches such as cIMS and SLIM remain diffusion limited, making separations exceedingly challenging when ΔCCS is less than approximately 0.5%.

Taken together, these complementary strengths suggest that combining chemical derivatization with IM-MS can simultaneously address the two principal analytical challenges in steroid analysis: limited ionization efficiency and limited structural discrimination. Historically, derivatizations reactions have largely been developed to improve analytical sensitivity, however covalent modification also alters molecular mass and gas-phase structure/conformation, suggesting that derivatization may fundamentally expand the structural information accessible from IM-MS measurements. Recognizing these complementary advantages, the combination of derivatization and IM-MS has recently emerged as a powerful analytical strategy to simultaneously separate, identify, and quantify steroid isomers in complex mixtures. This was first presented by Ahonen et al. using *p*-toluenesulfonyl isocyanate (PTSI) to derivatize hydroxyl groups in steroid epimers such as α/β-estradiol, androsterone/*epi-*androsterone, and testosterone/*epi*-testosterone.^21^ Subsequent work, primarily from our group, has focused on three chemical functionalities: hydroxyl group derivatization by carbonyl diimidazole (CDI);^57,58^ carbonyl derivatization by Girard’s reagents;^57,58^ and alkene reaction viz ozonolysis or the Paterno-Buchi reaction.^59,60^ In addition to various derivatization strategies,^61,62^ additional complementary approaches have included inducing isotopic shifts,^53^ adducting various cations/anions,^12,41,49,50,63–65^ and forming noncovalent complexes.^55,66,67^

Despite these advances, our previous studies have largely focused on improving the separation of specific steroid isomers; comparatively little attention has been devoted to understanding how derivatization chemistry systematically influences molecular mass, gas-phase structural diversity, and consequently IM-MS measurement space across multiple reagent classes. To investigate this concept in the present work, we focus on 11-oxosteroids, a subclass of adrenal hormones with clinical implications including as potential biomarkers for castration resistant prostate cancer (CRPC), congenital adrenal hyperplasia (CAH), polycystic ovary syndrome (PCOS), and other adrenal conditions.^68–70^ As their name implies, these 11-oxosteroids are androgen prohormones containing a C11 oxygen substituent (primarily carbonyl). For this reason, we selected a variety of hydrazine-based derivatization reagents (R-NH-NH_2_); we have previously demonstrated that this derivatization approach affects IM-MS behavior for anabolic androgenic steroids^58^ and endocrine hormones.^57^ By systematically evaluating multiple hydrazine-based derivatization chemistries across a common panel of 11-oxosteroids, this work establishes a systematic framework describing how chemical derivatization can be used to engineer IM-MS measurements through differential, yet controlled, manipulation of molecular mass, collision cross section, and gas-phase structural diversity.

## Experimental Section

### Chemicals and Reagents

Standards of 11-oxosteroids were provided by Advanced Stable Isotope (ASI) Chemicals (Cheyney, PA) and prepared as stock solutions (1 mg/mL) in methanol. Chemical formulas, molecular weights, and structures of all steroids are provided in Figure S1. Derivatization reagents (Girard’s Reagent D; Girard’s Reagent T; Girard’s Reagent P; 2,4-dinitrophenylhydrazine; dansyl hydrazine; 4-chlorophenylhydrazine; and 4-bromophenylhydrazine) were purchased from Fisher Scientific; their structures and other relevant chemical information are provided in Table S1. All solvents and other reagents were Fisher Scientific Optima LC-MS grade.

### Derivatization Reactions

Reactions were performed as previously for hydrazine-containing reagents.^57,58^ Briefly, 100 μL of a 1 mg/mL reagent solution in methanol was combined with 250 μL of methanol containing 10% acetic acid and 5 μL of a 1 mg/mL steroid stock solution. The reaction mixture was sonicated at room temperature for 15 min, dried under nitrogen, and reconstituted in 1 mL of 50:50 (v/v) water/methanol (0.1% formic acid) prior to IM-MS analysis. For MALDI analysis of in-solution reactions, a ground steel MTP 384 plate was first spotted with 2 μL of an α-cyano-4-hydroxycinnamic acid (CHCA) matrix solution in 30/70 water/methanol and allowed to dry. Next, 2 μL of the reaction mixture from above was spotted and allowed to dry. Finally, an additional 2 μL of CHCA solution was spotted on and allowed to dry. For on-surface reactions, the plate was first spotted with 2 μL of a CHCA solution in 30/70 water/methanol and allowed to dry before spotting 2 μL of the steroid stock solution. After drying, 2 μL of the reagent solution was spotted, followed by an additional 2 μL of CHCA solution. Scheme 1 shows the structures of a characteristic oxosteroid (adrenosterone), each of the seven derivatization reagents explored herein, and proposed reaction products (assuming reaction at the C3 carbonyl).

**Scheme 1:**
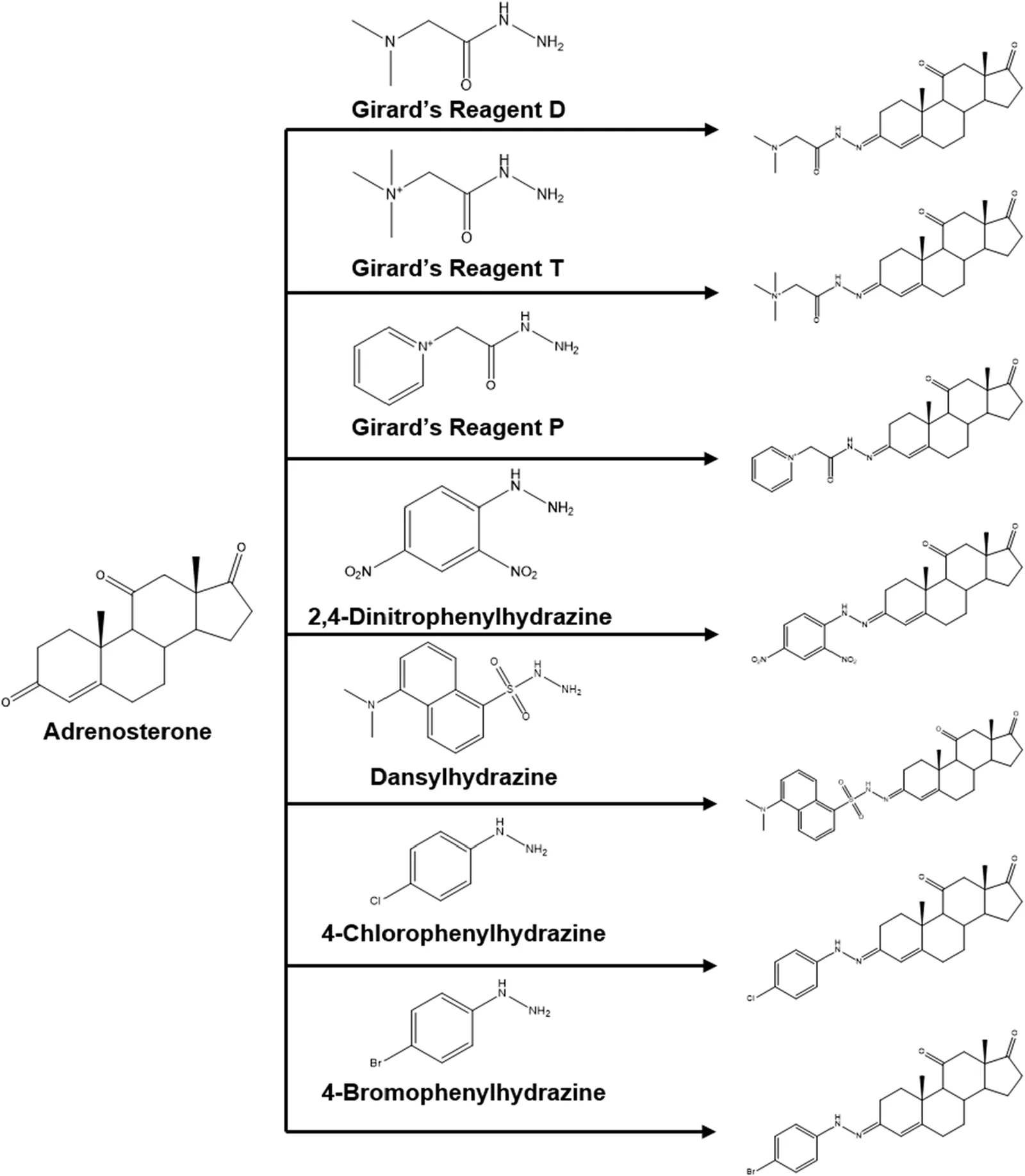
This Shows the Structures of a Characteristic Oxosteroid (Adrenosterone), Each of the Seven Derivatization Reagents Explored Herein, and Proposed Reaction Products (Assuming Reaction at the C3 Carbonyl)

### Mass Spectrometry Analyses

All IM-MS analyses were performed using either a 6560 drift tube IM-QTOF (Agilent Technologies, Santa Clara, CA) or MOBIE HRIM SLIM (MOBILion Systems, Inc., Chadds Ford, PA) coupled to Agilent 6546 QTOF. Both systems were equipped with an Agilent 1290 Infinity II UHPLC (Santa Clara, CA) operated at 0.300 mL/min (50:50 water/methanol with 0.1% formic acid) for flow injection analysis (FIA) of individual 10 μL injections. Ionization was performed with an Agilent Jetstream (AJS) electrospray ionization source in positive mode. For DTIM measurements, the drift tube was maintained at 3.95 Torr nitrogen gas and ambient temperature. DTIM separations were performed using a static electric field of 18.6 V/cm. Collision cross sections (CCS) were derived by the single field method using Agilent Tune Mix calibrants as described previously.^71^ Further details of the IM-QTOF parameters can be found in the Supporting Information (Table S2). For SLIM measurements, the SLIM device was maintained at 2.50 Torr nitrogen gas and ambient temperature. SLIM IM separations were performed using a separation TW of 35 kHz and 45 V_pp_; based on the electrode geometry, this frequency equates to a TW speed of 315 m/s. Raw CCS were derived and corrected as described previously.^72–74^ Further details of the SLIM and QTOF parameters can be found in the Supporting Information (Table S3). All MALDI-MS analyses were performed using a Bruker autoflex maX MALDI-TOF/TOF mass spectrometer equipped with a Smartbeam-II solid state laser (355 nm UV) for 500 laser shots.

Reaction product samples were analyzed over several days across the different instrument platforms (while stored in a refrigerator between experiments) with no significant change in product abundance and/or arrival time distributions. Subsequent analysis of the samples after a period of approximately 4 months of refrigerated storage did reveal significant degradation and/or undetectable products, indicating potential issues with long-term stability. While a full long-term stability study is outside of the scope of the present work, our recommendation would be to analyze samples immediately after reactions are performed (i.e., within a few days) and to store refrigerated at all times after preparation, including use of a refrigerated autosampler.

## Results and Discussion

### Engineering Ion Mobility-Mass Spectrometry (IM-MS) Measurement Space via Derivatization

One of the hallmark analytical benefits of IM-MS measurements is the hybrid separations afforded by the two techniques; in other words, mass spectrometry measures and separates based on mass-to-charge ratio (*m*/*z*), whereas ion mobility measures and separates based on size, shape, and charge (collectively mobility, K; or collision cross section, CCS). While differences in these measurands can afford improved analysis, especially for isomers/isobars (identical/similar *m*/*z*) that differ structurally and/or stereochemically, IM and MS are not truly orthogonal techniques; plotting CCS vs *m*/*z* generally results in positive correlations, especially within a given chemical class. This has been heralded as an advantage where biomolecules such as peptides, lipids, and carbohydrates are separated from one another in the two-dimensional CCS vs *m*/*z* space, but generally cluster tightly within a class.^75^ As such, a perpetual challenge in IM-MS has been pursuit of the maximum achievable peak capacity afforded by orthogonality. An excellent example of this pursuit has been via structural modification with covalent labeling; Fenn et al. derivatized carbohydrates with boronic acid in an effort to introduce molecular parachutes (i.e., larger increase in CCS relative to mass) or molecular anchors (larger increase in mass relative to CCS).^76^ One motivation of our present work was to build on this foundation by engineering IM-MS measurement space for steroids via derivatization, effectively changing mass and mobility differentially.

As proof of principle, we first performed derivatization of adrenosterone (11-oxoandrostenedione), with Girard’s Reagent D (GD), a small (MW 117) hydrazine-containing molecule with a dimethyl amine and no fixed charge (Scheme 1); this was selected as a representative reagent because it readily forms both mono- and doubly derivatized products while producing relatively uncomplicated IM-MS spectra. Analysis of the reaction mixture revealed a mass spectral base peak for the singly protonated [MGD + H]^+^ product at *m*/*z* 400.261. Other major peaks included: the singly charged double product, [MGD_2_ + H]^+^ at *m*/*z* 499.340; the doubly charged double product, [MGD_2_ + 2H]^+2^, at *m*/*z* 250.175; and unreacted Girard’s reagent, [GD + H]^+^ at *m*/*z* 118.098 (Figure 1A). Protonated and sodiated adrenosterone (expected at *m*/*z* 301.180 and 323.162, respectively) were only detected at a very low abundance, indicating the yield of the reaction was very high. Besides the mass addition afforded by the reaction, preliminary experiments using drift tube ion mobility (DTIM) showed the collision cross section (CCS) for the singly charged single product (213.1 Å^2^) was also increased relative to the unreacted protonated (173.4 Å^2^) and sodiated (198.2 Å^2^) species (Figure 1B). Also notable is the increased mobility peak width for the reaction product, suggesting the presence of unresolved features (i.e., conformers, geometric isomers, reaction site isomers, etc.) especially considering a slight shoulder was apparent on the high-mobility side of the peak. In this regard, adrenosterone is interesting because it contains C3, C11, and C17 carbonyls for potential reaction, and the observance of double products (both singly and doubly charged) in the mass spectrum indicates potential for the reaction to occur at two (or more) of these positions. To further investigate this, the reaction mixture was evaluated using high-resolution Structures for Lossless Ion Manipulations (SLIM) IM, which clearly demonstrated two mobility features at 211.0 and 213.5 Å^2^ (Figure 1C), indicating the presence of multiple gas-phase structural populations, the precise origin of which will be discussed below.

**Figure 1:**
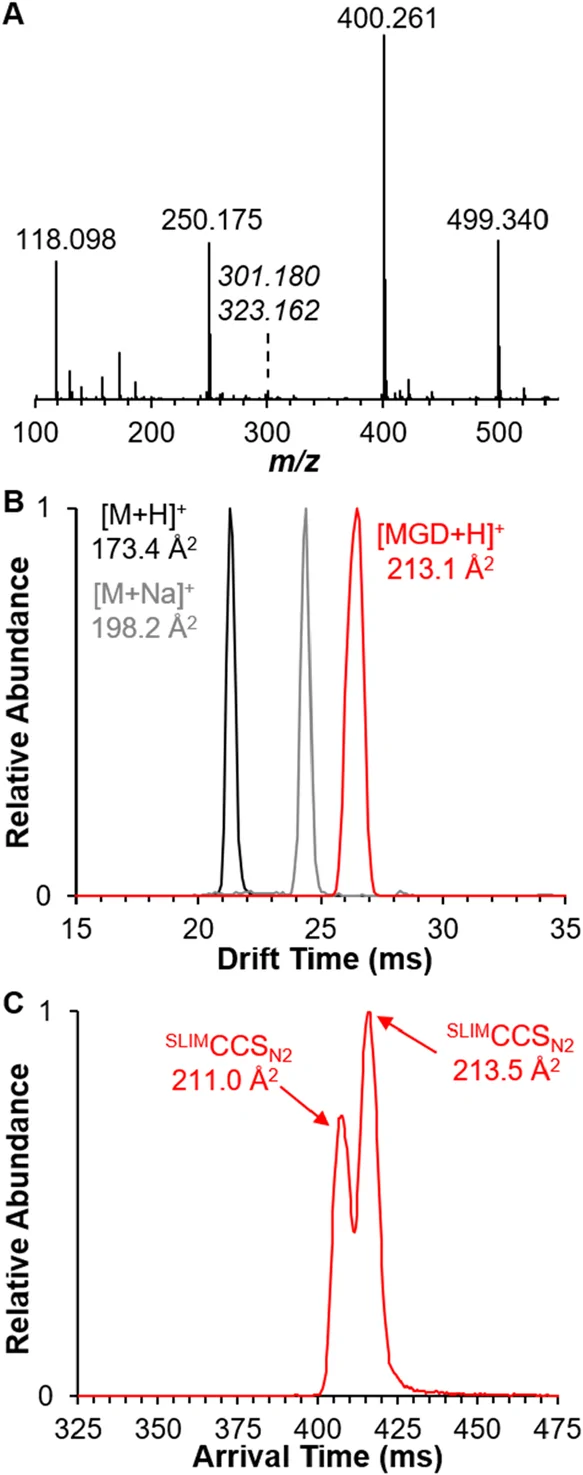
(A) Mass spectrum of the reaction mixture for adrenosterone with Girard’s Reagent D, featuring a base peak for [MGD + H]^+^ at *m*/*z* 400. Underivatized steroid is labeled at *m*/*z* 301 (protonated) and 323 (sodiated), as indicated by *italics.* (B) Comparison of drift tube ion mobility (DTIM) drift time and CCS for protonated/sodiated adenosterone relative to the major derivatization product. (C) SLIM IM measurement of [MGD + H]^+^ reveals two different mobility features, presumably derivatization location isomers.

We next explored six additional hydrazine-containing reagents: Girard’s Reagents T (GT) and P (GP); dinitrophenylhydrazine (DNPH); dansyl hydrazine (Dh); and 4-chloro-and 4-bromophenylhydrazine. Although all reagents increased both molecular mass and CCS, the magnitude of these changes relative to unreacted adrenosterone depended strongly on reagent chemistry (Figure 2A). As a systematic study in how these mass/mobility shifts differed from the trend for this biomolecular class, we applied these reagents to 16 different 11-oxosteroids. Figure 2B shows CCS vs *m*/*z* trends for the protonated/sodiated unreacted steroids and all seven sets of (singly charged) single reaction products. The Girard’s reagents, being small organic compounds, added both mass and CCS to the steroids, but they generally clustered within the expected space for the unreacted (sodiated) species. However, we observed that reagents with higher-density substituents (e.g., chlorine and bromine) or with much larger mass additions (e.g., DNPH and Dh) both shifted the clusters away from the expected occupied space for the sodiated unreacted steroids; in contrast to molecular “parachutes”, which primarily increase CCS, the derivatization chemistries investigated here generally behaved as molecular “anchors” by producing disproportionately large increases in molecular mass relative to CCS. For example, derivatization by Girard’s Reagent D introduces a roughly 32% increase in mass with only a 14–22% increase in CCS, while derivatization by dansyl hydrazine introduces a > 80% increase in mass with only a 17–32% increase in CCS (Table S4). Tables S5–S11 provide *m*/*z* and CCS for major reaction products across the surveyed steroids and derivatization reagent.

**Figure 2:**
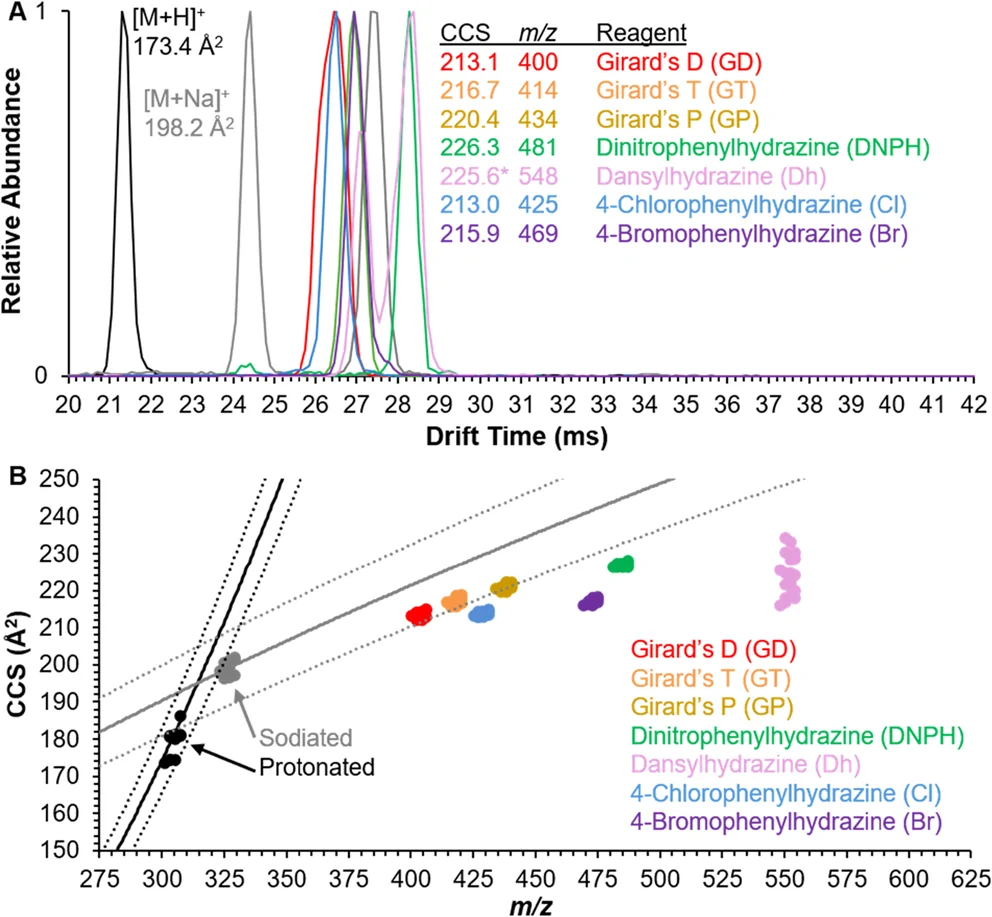
(A) Comparison of mobility for protonated/sodiated unreacted adrenosterone with the major product for each of seven derivatization reactions. The dansyl hydrazine-derivatized product yielded two mobility features; the CCS listed is for the major feature (as denoted by asterisk). (B) Mobility vs mass trend plots for the major product of 16 steroids with the seven derivatization reactions. Trend lines for protonated/sodiated (solid lines) are supplemented by upper and lower bounds (±5%) as indicated by the dashed lines.

Importantly, rather than uniformly translating all products through CCS vs *m*/*z* space, each derivatization chemistry generated a characteristic shift reflecting its unique influence on molecular mass and gas-phase structure; this provides a means to systematically and controllably reposition analytes within the IM-MS measurement space. Nevertheless, these reagent-dependent shifts do not fully capture the conformational information encoded within the individual mobility distributions. As discussed below, several derivatization chemistries also generated characteristic mobility fingerprints that substantially increased the structural information available from IM-MS measurements.

### Structural Information Encoded in IM-MS Measurement of Derivatization Products

As we have demonstrated previously with IM-MS analysis of derivatized steroids, these mobility fingerprints are highly dependent on both the structure of the steroid and the chemistry of the reagent.^57–60^ The series of 16 oxosteroids investigated in this study (encompassing several isomer groups, as shown in Figure S1) were selected due to relevant differences in their C3/C11/C17 hydroxyl/carbonyl compositions. By performing derivatization using Girard’s Reagent D, we observed that the [MGD + H]^+^ product was present as the mass spectral base peak in nearly all product solutions; no reaction products were detected for 11-ketoandrosterone, 11-keto-5α-androstanediol, or 11β-hydroxyandrostanediol. The lack of product for 11β-hydroxyandrostanediol was unsurprising, as this -triol compound contains C3, C11, and C17 alcohols (no carbonyls present for reaction). However, the two other compounds both contain a C11 carbonyl, and 11-ketoandrosterone also contains a C17 carbonyl. In addition to the single reaction product, each compound from the first set of isomers (MW 302) produced double reaction products ([MGD_2_ + H]^+^ at *m*/*z* 501) except for 11-ketotestosterone; these patterns appeared to demonstrate a preferential derivatization at the C3 carbon, with secondary reaction occurring at C17. This general trend continued across all 16 steroids surveyed via Girard’s Reagent D: single reaction product was observed for all C3=O-containing species; double products (singly and doubly protonated) were observed for all C3=O/C17=O-containing species; and the identity of the C11 substituent did not appear to affect the derivatization chemistry whatsoever. The latter observation was quite interesting as our original rational for focusing on hydrazine derivatization chemistry was to exploit the additional carbonyl group (at C11) in most of these oxosteroids.

Next, derivatization was performed with Girard’s Reagents T and P, both containing a fixed charge quaternary amine. As with the Girard’s D reaction, double products were observed for all compounds containing both C3 and C17 carbonyls. While minimal separation was revealed for these species using low-resolution DTIM (Figure 3A,B), SLIM measurement did provide an interesting observation: those compounds lacking a C17 carbonyl group, and therefore unable to produce double products, all displayed a bimodal mobility distribution for their Girard T-derivatized single reaction product, while those with two carbonyl groups had a monomodal distribution (Figure 3C). Because 11β-hydroxytestosterone only contains a single carbonyl (thus cannot form reaction site isomers) and a fixed charge on Girard’s Reagent (therefore unlikely to produce charge site isomers), the mobility distribution is consistent with either E/Z hydrazone isomers or conformational isomers. Unfortunately, the mobility-aligned MS/MS patterns for these two features did not show any significant differences. Interestingly, the same behavior was not observed for products of Girard’s Reagent P (Figure 3D) where all single products yield a monomodal distribution, indicating that the fixed charge on the relatively flexible side chain of GT results in a different charge distribution (and thus different conformational space) than the delocalized charge on the pyridinium ring of GP. Alternatively, the potential exists that both products yield E/Z hydrazone isomers that (in the case of GP) are unresolved even with high-resolution SLIM measurements. Collectively, these observations demonstrate that derivatization generates structure-dependent mobility fingerprints that extend beyond a single CCS value; these fingerprints incorporate the number, relative abundance, and CCS of mobility-resolved structural populations, providing richer structural information than molecular mass or CCS alone.

**Figure 3:**
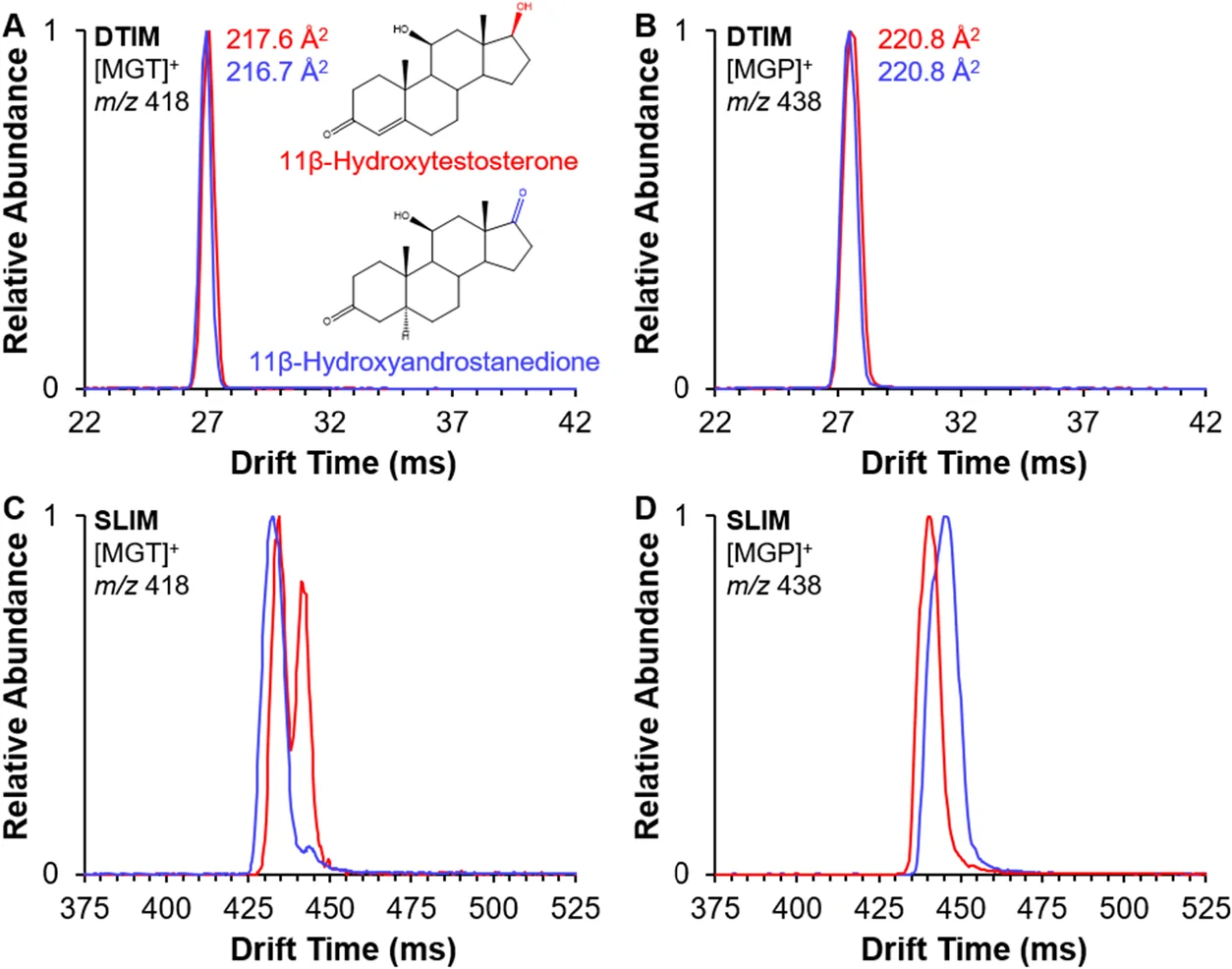
Reaction of 11β-hydroxytestosterone and 11β-hydroxyandrostanedione show monomodal mobility distributions when measured with DTIM for both (A) GT- and (B) GP-derivatized single products. However, SLIM IM analysis reveals (C) a bimodal mobility distribution for the GT product of 11β-hydroxytestosterone, yet (D) still a monomodal mobility distribution for both GP products.

Interestingly, across our survey of the several derivatization reagents only monoderivatization was consistently observed for the aryl hydrazine reagents (DNPH, 4-bromophenylhydrazine, and 4-chlorophenylhydrazine), whereas doubly derivatized products were readily formed with all four hydrazide-based reagents (Girard’s D/T/P and dansyl hydrazine). This pattern suggests that the formation of doubly derivatized products is governed not only by the presence of multiple carbonyl groups within the steroid, but also by the intrinsic reactivity of the derivatization reagent under the reaction conditions employed.

The most structurally informative reagent was dansyl hydrazine, which produced unique and distinctive mobility fingerprints across several different product ions. For example, adrenosterone’s singly charged single product ([MDh + H]^+^ at *m*/*z* 548) yielded two distinct features, while its singly charged double product ([MDh_2_ + H]^+^ at *m*/*z* 795) yielded only one mobility feature, and its doubly charged double product ([MDh_2_ + 2H]^+2^ at *m*/*z* 398) yielded three separate features (Figure 4A). Furthermore, within MW 304 isomer group each of five compounds displayed unique CCS and/or fingerprints (Figure 4B): (i) 11β-hydroxytestosterone displays a bimodal mobility distribution with features at 222.7 and 229.3 Å^2^; (ii) 11β-hydroxyandrostanedione displays a trimodal distribution with features at 218.5, 224.3, and 233.3 Å^2^; (iii) 11-keto-5α-dihydrotestosterone displays only a monomodal distribution with feature at 224.6 Å^2^; (iv) 11-keto-5β-dihydrotestosterone displays a bimodal distribution with features at 221.7 and 228.4 Å^2^; and (v) 11β-hydroxy-5β-androstane-3,17-dione displays only a monomodal distribution with feature at 218.8.6 Å^2^. Finally, we demonstrated that subtle structural features detected in low-resolution DTIM (Figure 4C) can be more comprehensively profiled by the high-resolution afforded by SLIM (Figure 4D). These observations demonstrate that the additional resolving power of SLIM does not fundamentally alter the mobility fingerprints generated by derivatization; rather, it reveals structural populations that are only partially resolved by conventional DTIM. Thus, while our first results section demonstrated that derivatization systematically shifts IM-MS measurement space, these results further show that individual derivatization chemistries encode additional structural information within the mobility distributions themselves. These characteristic mobility fingerprints provide a substantially richer description of derivatized steroids than CCS alone and establish the foundation for the enhanced analytical discrimination discussed in the following section.

**Figure 4:**
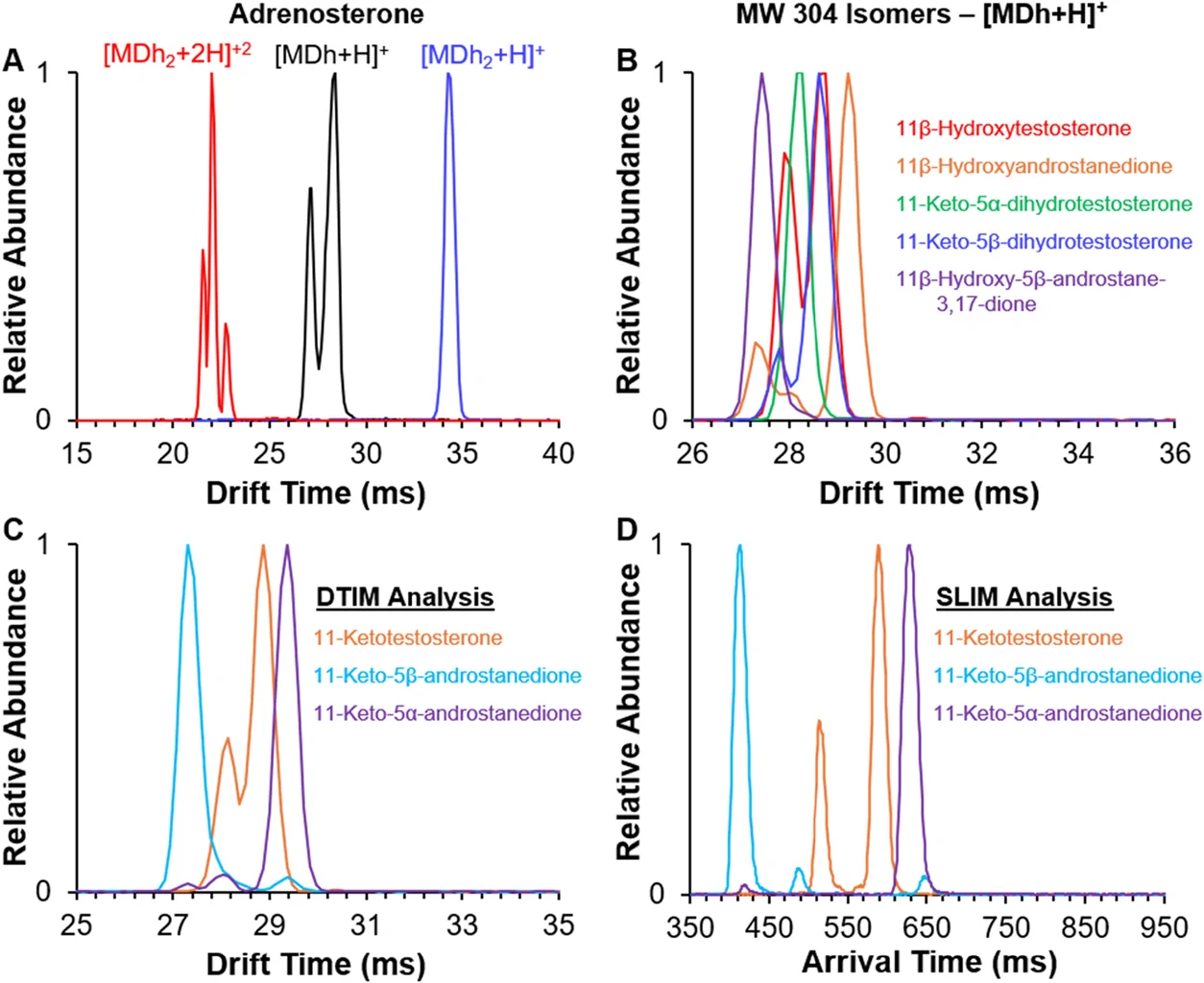
(A) Dansyl hydrazine-derivatized adrenosterone shows unique mobility fingerprints for single product, singly charged double product, and doubly charged double product. (B) Each of the five MW 304 isomers show unique mobility fingerprint and/or CCS. (C) DTIM analysis of dansyl hydrazine-derivatized MW 302 isomers shows unique fingerprints, which are amplified by (D) high-resolution SLIM analysis.

### Improved Steroid Differentiation through Derivatization and IM-MS

Previous studies from our group demonstrated that targeted derivatization can enhance mobility differences among selected anabolic steroids and endocrine hormones.^57–60^ However, those investigations focused on a limited number of steroid pairs and individual derivatization chemistries. While our previous results herein have demonstrated that derivatization systematically changes IM-MS measurements and generates characteristic mobility fingerprints, the analytical value of these observations depends on whether they improve differentiation of structurally similar steroids. We therefore evaluated the ability of these derivatization strategies to enhance analytical discrimination across the panel of 16 11-oxosteroids. Interestingly, despite the relative simple derivatization pattern using the Girard’s Reagent D, separation was achievable for many of the isomers surveyed herein based on different reaction products and/or different IM-MS techniques. For example, isomer pairs 11-keto-5β-androstanedione/11-keto-5α-androstanedione (Figure 5A) and 11β-hydroxyandrostanedione/11β-hydroxy-5β-androstane-3,17-dione (Figure 5B) were well resolved with traditional DTIM as their [MGD_2_ + H]^+^ products. Although isomer pairs 11β-hydroxytestosterone/11β-hydroxyandrostanedione and 11β-hydroxyDHT/11β-hydroxyandrosterone remained unresolved as their [MGD + H]^+^ products by conventional DTIM, the increased resolving power afforded by SLIM completely differentiated the products (Figure 5C,D). Derivatization by 4-chloro- and 4-bromophenyl hydrazine provided some similar isomer separations to Girard’s Reagent D, but their contribution was important based on mass: both chlorine and bromine provide unique M + 2 isotopic patterns that allow ready identification of reaction products (Figure S2), not just in these targeted studies but also potentially in untargeted studies where low abundance reaction products may be masked by interferences. The analytical contribution of these reagents therefore extends beyond mobility separation; their characteristic isotopic signatures provide an additional orthogonal descriptor that may facilitate confident recognition of derivatization products, particularly in untargeted workflows where low-abundance products may otherwise be obscured by chemical background. More importantly, this provides an avenue by which to introduce potentially unique patterns for reagents selectively targeting different functional groups. More broadly, these observations illustrate that derivatization reagents can be rationally designed to contribute multiple complementary analytical descriptors including mobility shifts, characteristic fingerprints, reaction stoichiometry, and isotopic signatures for enhanced molecular identification.

**Figure 5:**
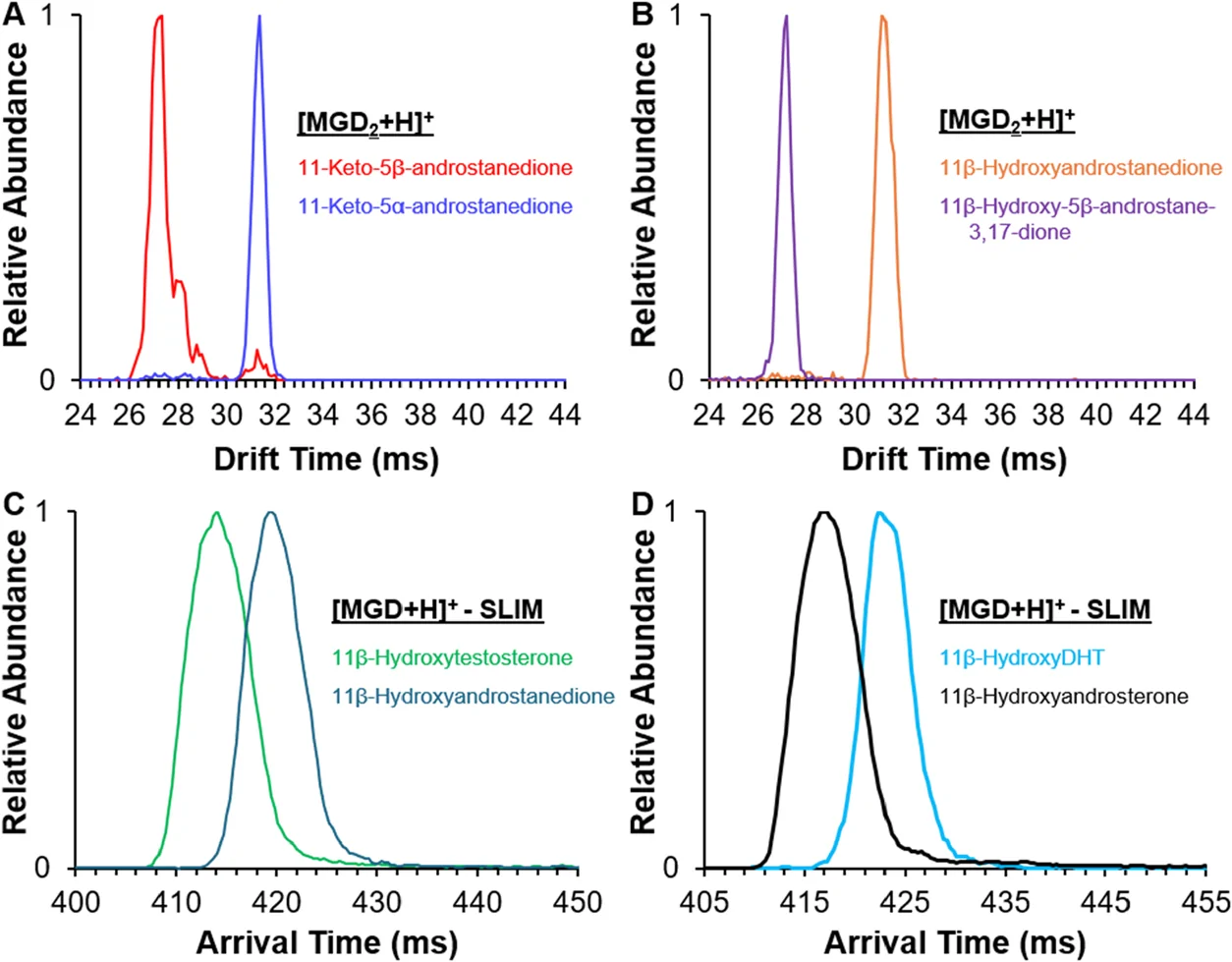
DTIM achieved separation of [MGD_2_ + H]^+^ reaction products for isomer pairs (A) 11-keto-5β-androstanedione/11-keto-5α-androstanedione, and (B) 11β-hydroxyandrostanedione/11β-hydroxy-5β-androstane-3,17-dione. SLIM achieved separation of [MGD + H]^+^ products for isomer pairs (C) 11β-hydroxytestosterone/11β-hydroxyandrostanedione, and (D) 11β-hydroxyDHT/11β-hydroxyandrosterone.

### Evaluating Alternative Ionization Techniques and Future Potential

To this point, steroid derivatization has largely been performed in bulk solution for analysis by IM-MS with ESI and/or APCI. We next investigated whether these chemistries could be translated to alternative ionization methods and ultimately to surface-based reaction environments, specifically using matrix-assisted laser desorption/ionization (MALDI) in this case. We first performed in-solution reactions of 11β-hydroxyandrostenedione with the seven previously discussed derivatization reagents and then spotted the resulting product mixture on a MALDI plate (coated with CHCA matrix) for analysis; Figure 6A,B shows representative mass spectra for in-solution reactions with Girard’s Reagents T and P. These spectra revealed similar products as observed via ESI and confirmed by MS/MS; for example, major fragments for [MGT]^+^ at *m*/*z* 357 and 267 match the ESI-MS spectra acquired on the DTIM-QTOF. This demonstrates that the derivatization strategies developed herein appear transferrable to other ionization techniques, in this case MALDI. Next, we performed derivatization using 4-bromophenylhydrazine on all 16 steroids and again observed similar products to ESI. This shows that the reactions are broadly available across the range of 11-oxosteroids surveyed in this study.

**Figure 6:**
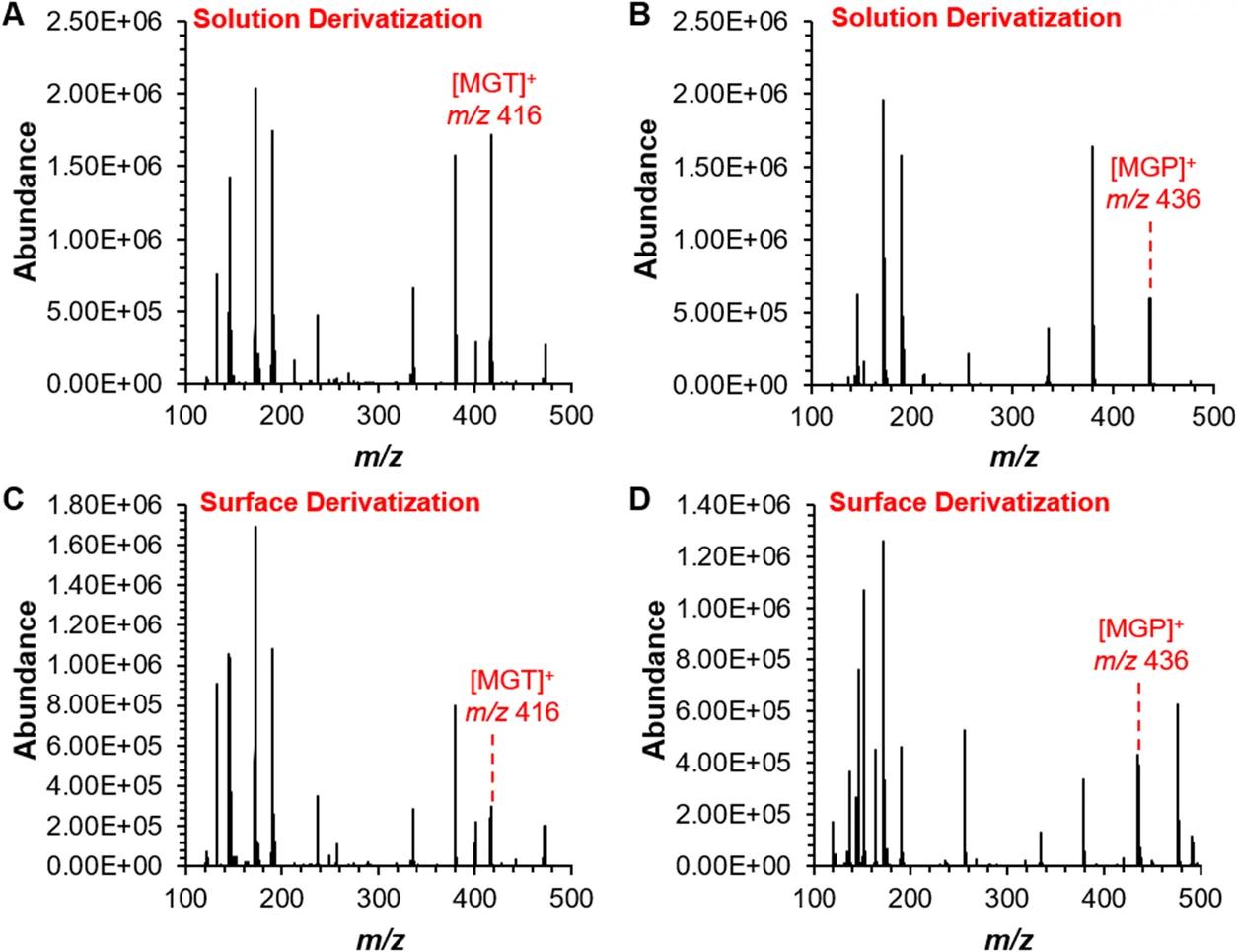
Adrenosterone was reacted in-solution with (A) Girard’s Reagent T and (B) Girard’s Reagent P and then spotted on a MALDI plate for analysis. The resulting spectra show similar products to those observed in ESI-MS. Next, adrenosterone was spotted directly onto a MALDI plate, and then (C) Girard’s Reagent T and (D) Girard’s Reagent P were spotted on the dried steroid. MALDI analysis again revealed similar products, indicating this reaction scheme works “on-surface”.

Having established compatibility with MALDI analysis, we next investigated whether these derivatization reactions could be performed directly on analytical surfaces, potential application to on-tissue chemical derivatization and mass spectrometry imaging (MSI). Traditional MSI workflows lack a preseparation step (i.e., chromatography) and therefore can be challenged with differentiating isomers, even when implementing MS/MS. While there have been numerous reports of coupling MSI and IMS,^77–80^ the incorporation of on-surface chemistry to improve mobility separation and structural characterization has been limited.^47,81,82^ As a proof of principle, we spotted 11β-hydroxyandrostenedione on a MALDI plate and, after allowing it to dry, then spotted solutions each containing an individual derivatization reagent. After applying matrix, MALDI analysis again revealed the same products as previously detected by ESI-MS and MALDI-MS of the in-solution reactions (Figure 6C,D). Notably, these reactions were successfully performed without extensive optimization of on-surface reaction conditions, suggesting that hydrazine derivatization is inherently compatible with surface-based analytical workflows. Furthermore, derivatization may simultaneously improve MALDI analyses through increased ionization efficiency, reduced interference from low-mass matrix ions, and enhanced structural discrimination by IM-MS/MS. Collectively, these experiments demonstrate that the derivatization chemistry is remarkably robust to changes in both ionization modality and reaction environment, and this approach shows promise for future utility in a combined MALDI MSI/IM-MS workflow including on-surface (or on-tissue) chemical derivatization.

## Conclusions

Chemical derivatization has traditionally been viewed primarily as a means to improve ionization efficiency and analytical sensitivity in mass spectrometry. The results presented here demonstrate that its analytical value extends well beyond sensitivity enhancement. By systematically evaluating seven hydrazine-based derivatization chemistries across a panel of 16 isomeric 11-oxosteroids, we show that covalent modification fundamentally alters the information content accessible by IM-MS. First, derivatization systematically redistributed steroids throughout CCS vs *m*/*z* measurement space, with each reagent producing characteristic shifts that reflected its unique influence on molecular mass and gas-phase structure. Rather than uniformly translating analytes, individual reagent classes engineered distinct regions of IM-MS measurement space (i.e., mass vs mobility). Second, high-resolution IMS measurements revealed that many derivatization products encoded characteristic mobility fingerprints composed of multiple mobility-resolved structural populations. While it should be noted that generation of multiple mobility features could potentially impact sensitivity, these fingerprints provided substantially richer structural information than molecular mass or CCS alone and demonstrated that derivatization chemistry directly influences gas-phase conformational diversity. Third, these measurements translated into improved analytical discrimination of structurally similar steroids through complementary descriptors including CCS, reaction stoichiometry, mobility fingerprints, and reagent-specific isotopic signatures. Finally, successful translation of these chemistries to MALDI analysis, including direct surface derivatization, demonstrates that these concepts are not restricted to solution-phase electrospray workflows and provides a foundation for future integration with MALDI imaging experiments. Collectively, these results establish chemical derivatization as a general strategy for engineering the analytical information accessible by IM-MS. By systematically manipulating molecular mass, CCS, and gas-phase structural diversity, derivatization provides a versatile framework for simultaneously enhancing analytical sensitivity and structural discrimination across a broad range of future IM-MS applications. In future studies, we aim to apply these derivatization methods (especially using dansyl hydrazine, which presented the most promising results in the current study) to untargeted characterization of steroids, and ultimately the metabolome as a whole, in biological samples.

## Supplementary Material



## Notes

Structure of all derivatization reagents; 6560 IM-QTOF and SLIM IM-MS instrumental parameters; CCS values for all major reaction products of all steroids/derivatization reagents; structure of all 11-oxosteroids; and reaction scheme (PDF)
