# Supplementary material for: Expanding the Measurement Space of Ion Mobility-Mass Spectrometry Using Chemical Derivatizations
Source: ACS Meas Sci Au. 2026 Aug 8;6(5):1825–36. doi: 10.1021/acsmeasuresciau.6c00210 (PMC13693263; doi:10.1021/acsmeasuresciau.6c00210)
Supplement: Supplementary file 1 [file tg6c00210_si_001.pdf]

SUPPORTING INFORMATION FOR:

Expanding the Measurement Space of Ion Mobility-Mass Spectrometry using Chemical Derivatizations

*Sabrina Fernandez, Angela Acateca, Bradley B. Garrison, Heidi M. Sabatini, Christopher D. Chouinard**

Clemson University, Department of Chemistry; Clemson, SC, USA 29634

Corresponding author email: cchouin@clemson.edu

KEYWORDS: Steroids; Derivatization; Ion Mobility-Mass Spectrometry; Structures for Lossless Ion Manipulations (SLIM); Tandem Mass Spectrometry

Table of Contents

Table S1. Structure and details of all derivatizing reagents……………………………………………S2
Table S2. Instrumental parameters for the Agilent 6560 IM-QTOF....……………………………..S3
Table S3. Instrumental parameters for the MOBILion MOBIE SLIM & Agilent 6546 QTOF..........S4
Table S4. CCS values, Girard's Reagent D Products……..…………………………………..……..S6
Table S5. CCS values, Girard's Reagent T Products….………………..……………………………S6
Table S6. CCS values, Girard's Reagent P Products…..…………………...…………………………S6
Table S7. CCS values, 2,3-Dinitrophenylhadrazine Products………..…...…………………………S7
Table S8. CCS values, Dansyl Hydrazine Products……………………...……………………………S7
Table S9. CCS values, 4-Chlorophenylhydrazine Products...……………..…………………………S7
Table S10. CCS values, 4-Bromophenylhydrazine Products……………..………………………S8
Figure S1. 11-oxosteroids: chemical formula, molecular weight, and structure…………………..S9
Figure S2. Mass spectra for Cl/Br-containing products..…………………………. ……………...S13

SUPPORTING TABLES

Table S1. Structure and details of all derivatizing reagents.

Reagent Name	Structure	Chemical Formula	Molecular Weight
Girard's Reagent D	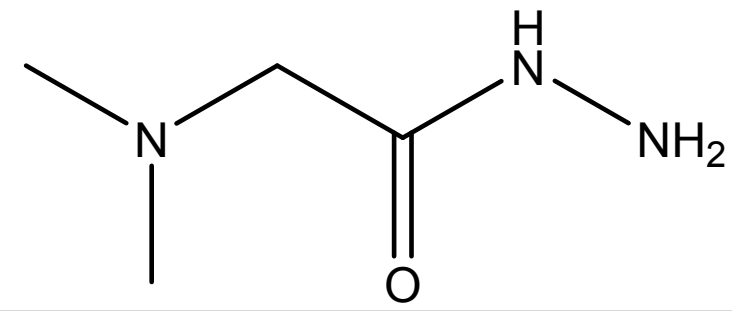	$C_4H_{11}N_3O$	117.15
Girard's Reagent T	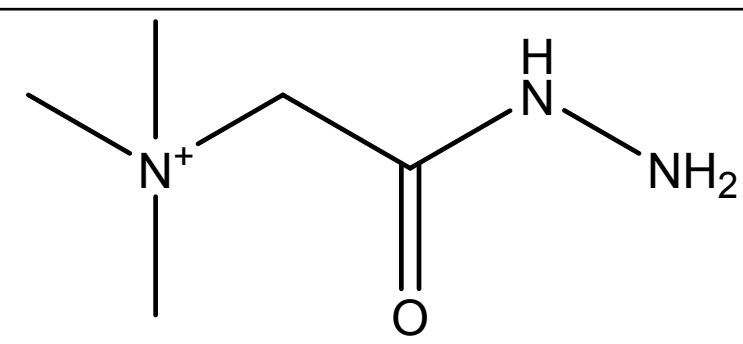	$C_5H_{14}N_3O$	132.18
Girard's Reagent P	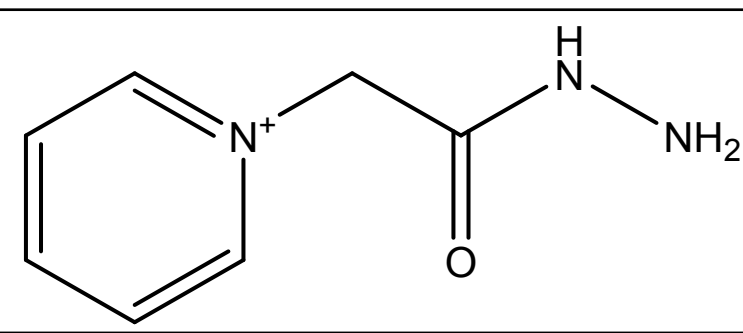	$C_7H_{10}N_3O$	152.17
2,4-Dinitrophenyl hydrazine	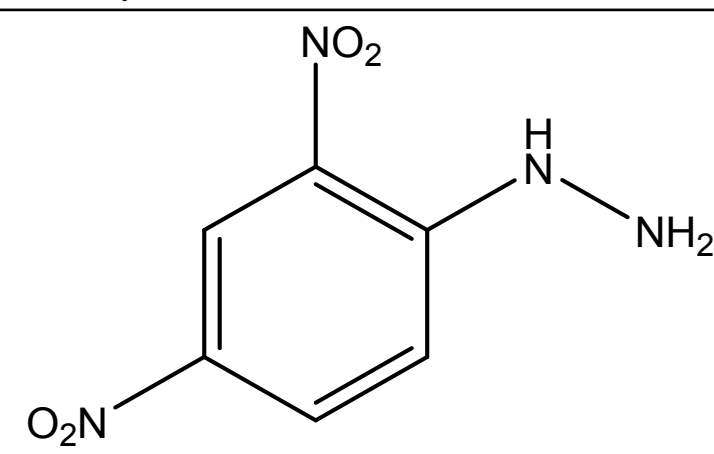	$C_6H_6N_4O_4$	198.14
Dansyl Hydrazine	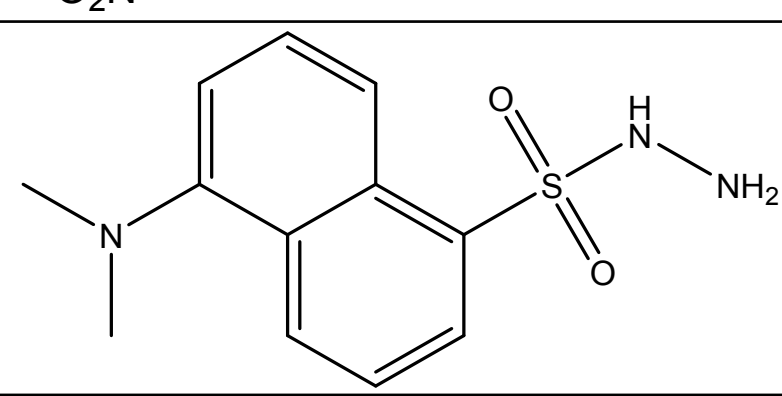	$C_{12}H_{15}N_3O_2S$	265.33
4-Chlorophenyl hydrazine	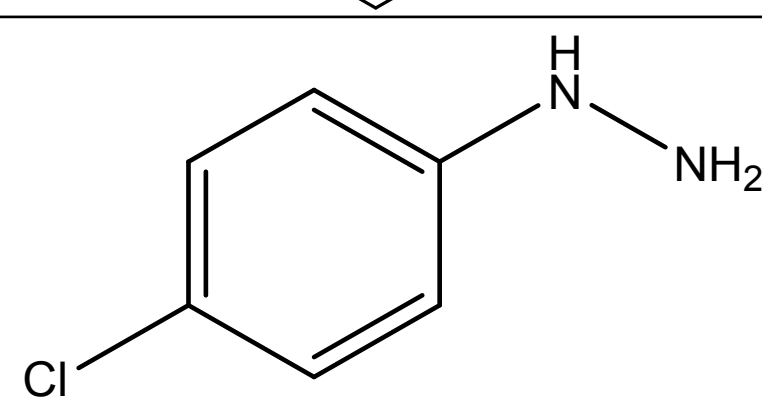	$C_6H_7N_2Cl$	142.59
4-Bromophenyl hydrazine	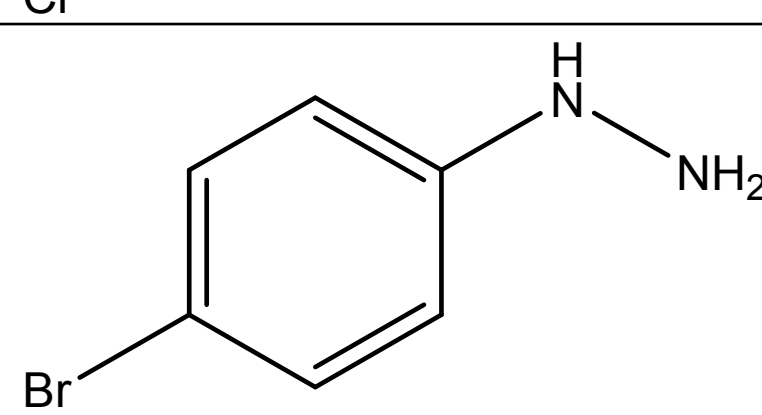	$C_6H_7N_2Br$	187.04

Table S2. Instrumental parameters for the Agilent 6560 IM-QTOF.

Instrument Region	Instrumental Parameter	Experimental Value
**Source** Agilent AJS ESI	Gas Temp	325 °C
	Drying Gas	12 L/min
	Nebulizer	20 psi
	Sheath Gas Temp	275 °C
	Sheath Gas Flow	10 L/min
	VCap	4000 V
	Nozzle Voltage (Expt)	1000 V
	Fragmentor	400 V
	Oct 1 RF Vpp	750 V
**High Pressure Funnel**	High Pressure Funnel Delta	150 V
	High Pressure Funnel RF	200 V
**Trapping Funnel**	Trap Funnel Delta	180 V
	Trap Funnel RF	200 V
	Trap Funnel Exit	10 V
	Entrance Grid Delta	10 V
	Entrance Grid Low	96 V
	Entrance Grid High	107 V
	Trap Entrance	91 V
	Trap Exit	90 V
	Trap Fill Time	5000 μs
	Trap Release Time	150 μs
	Trap Exit Grid 1 Delta	4 V
	Trap Exit Grid 1 Low	86.3 V
	Trap Exit Grid 1 High	90.3 V
	Trap Exit Grid 2 Delta	8.5 V
	Trap Exit Grid 2 Low	85.8 V
	Trap Exit Grid 2 High	94.3 V
**Drift Tube**	Drift Tube Entrance Voltage	1700 V
	Drift Tube Exit Voltage	250 V
	Drift Tube Field Strength	18.6 V/cm

Table S3. Instrumental parameters for the MOBILion MOBIE SLIM & Agilent 6546 QTOF.

**Instrument Region**	**Instrumental Parameter**	**Experimental Value**
**Ionization Source**<br>Agilent AJS ESI	Polarity	Positive
	Gas Temp	325 °C
	Vaporizer	200 °C
	Drying Gas	8 L/min
	Nebulizer	45 psi
	VCap	4000 V
	Charging Voltage (Expt)	2000 V
	Fragmentor	175 V
	Skimmer	45 V
	Oct 1 RF Vpp	750 V
**SLIM Conditions**	Funnel In	165 V
	Funnel Out	100 V
	Funnel Conductance Limit (CL)	95 V
	SLIM Bias	90 V
	SLIM Mode	HRIM (13 m)
	SLIM Wave Shape	Sine
	Fill Time	100 ms
	Trap Time	0.3 ms
	Release Time	5.2 ms
	IMS Frame Length	1200 ms
	Fill TW Frequency	15 kHz
	Fill TW Amplitude	5 V_{pp}
	Release TW Frequency	20 kHz
	Release TW Amplitude	25 V_{pp}
	Separation TW Frequency	35 kHz
	Separation TW Speed	315 m/s

	Separation TW Amplitude	45 V_{pp}
	SLIM Exit CL	50 V
	Quad Bias	45 V
	Quad Pressure (Rough Vac)	2.5 Torr
	SLIM RF Amplitude	220 V
	SLIM RF Frequency	1200 kHz
	SLIM Board Spacing	3 mm

Table S4. Increases in mass and CCS (by percentage) for each of the protonated reaction products (or fixed charge, for Girard's Reagents T and P) relative to the protonated native steroid.

Compound	$[MGD+H]^+$ *m/z*	$[MGD+H]^+$ $^{DT}CCS_{N2}$	$[MGT]^+$ *m/z*	$[MGT]^+$ $^{DT}CCS_{N2}$	$[MGP]^+$ *m/z*	$[MGP]^+$ $^{DT}CCS_{N2}$
**Adrenosterone**	+32.9%	+22.9%	+37.6%	+25.0%	+44.2%	+27.1%
**11β-Hydroxyandrostendione**	+32.7%	+22.2%	+37.3%	+24.3%	+43.9%	+26.6%
**11-Ketotestosterone**		+21.7%		+24.3%		+26.6%
**11-Keto-5β-androstanedione**		+18.4%		+19.3%		+21.4%
**11-Keto-5α-androstanedione**		+18.6%		+20.1%		+22.3%
**11β-Hydroxytestosterone**	+32.5%	+21.7%	+37.1%	+24.9%	+43.6%	+26.7%
**11β-Hydroxyandrostanedione**		+17.6%		+19.6%		+21.9%
**11-Keto-5α-dihydrotestosterone**		+18.8%		+21.1%		+23.1%
**11-Keto-5β-dihydrotestosterone**		+18.8%		+21.4%		+23.3%
**11β-Hydroxy-5β-androstane-3,17-dione**		+17.6%		+19.1%		+21.2%
**11β-Hydroxydihydrotestosterone**	+32.2%	+18.7%	+36.8%	+20.9%	+43.3%	+22.7%
**11β-Hydroxy-5β-dihydrotestosterone**		+18.9%		+20.4%		+22.4%
**11β-Hydroxyandrosterone**		+14.4%		+16.3%		+18.5%

Compound	$[MDNPH+H]^+$ *m/z*	$[MDNPH+H]^+$ $^{DT}CCS_{N2}$	$[MDh+H]^+$ *m/z*	$[MDh+H]^+$ $^{DT}CCS_{N2}$
**Adrenosterone**	+59.8%	30.5%	+82.0%	+24.6-30.1%
**11β-Hydroxyandrostendione**	+59.4%	30.4%	+81.5%	+24.4-27.2%
**11-Ketotestosterone**		30.4%		+28.8-32.1%
**11-Keto-5β-androstanedione**		25.1%		+20.5%
**11-Keto-5α-androstanedione**		25.5%		+29.8%
**11β-Hydroxytestosterone**	+59.0%	30.4%	+81.0%	+27.8-31.6%
**11β-Hydroxyandrostanedione**		25.5%		+20.6-28.8%
**11-Keto-5α-dihydrotestosterone**		25.9%		+24.4%
**11-Keto-5β-dihydrotestosterone**		26.1%		+23.1-26.8%
**11β-Hydroxy-5β-androstane-3,17-dione**		24.9%		+20.8%
**11β-Hydroxydihydrotestosterone**	+58.6%	26.0%	+80.4%	+23.8%
**11β-Hydroxy-5β-dihydrotestosterone**		25.7%		+21.6-26.5%
**11β-Hydroxyandrosterone**		21.5%		+17.0-23.6%

Compound	$[MCl+H]^+$ *m/z*	$[MCl+H]^+$ $^{DT}CCS_{N2}$	$[MBr]^+$ *m/z*	$[MBr]^+$ $^{DT}CCS_{N2}$
**Adrenosterone**	+41.2%	+22.8%	+55.8%	24.5%
**11β-Hydroxyandrostendione**	+40.9%	+22.2%	+55.4%	24.4%
**11-Ketotestosterone**		+22.2%		24.4%
**11-Keto-5β-androstanedione**		+17.8%		19.6%
**11-Keto-5α-androstanedione**		+18.8%		20.3%
**11β-Hydroxytestosterone**	+40.6%	+23.0%	+55.0%	24.4%
**11β-Hydroxyandrostanedione**		+18.3%		20.3%
**11-Keto-5α-dihydrotestosterone**		+18.8%		20.7%
**11-Keto-5β-dihydrotestosterone**		+18.7%		20.4%
**11β-Hydroxy-5β-androstane-3,17-dione**		+17.6%		19.2%
**11β-Hydroxydihydrotestosterone**	+40.4%	+18.6%	+54.7%	20.5%
**11β-Hydroxy-5β-dihydrotestosterone**		+18.6%		20.5%
**11β-Hydroxyandrosterone**		+14.6%		16.4%

Table S5. $^{DT}CCS_{N2}$ and *m/z* values for all Girard's Reagent D derivatization major reaction products.

Compound	[MGD+H]$^+$ *m/z*	$^{DT}CCS_{N2}$ (Å^2)	[MGD$_2$+H]$^+$ *m/z*	$^{DT}CCS_{N2}$ (Å^2)	[MGD$_2$+2H]$^{+2}$ *m/z*	$^{DT}CCS_{N2}$ (Å^2)
**Adrenosterone**	400.259	213.1 ± 0.1	499.341	248.2 ± 0.1	250.174	291.3 ± 1.1
**11β-Hydroxyandrostendione**	402.275	213.1 ± 0.1	501.357	248.2 ± 0.1	251.182	290.6 ± 0.1
**11-Ketotestosterone**		212.1 ± 0.1		ND		ND
**11-Keto-5β-androstanedione**		214.1 ± 0.1		218.1 ± 0.6		292.6 ± 0.1
**11-Keto-5α-androstanedione**		214.1 ± 0.1		251.1 ± 0.1		292.6 ± 0.1
**11β-Hydroxytestosterone**	404.291	212.1 ± 0.1	503.373	ND	252.190	ND
**11β-Hydroxyandrostanedione**		213.1 ± 0.1		249.4 ± 0.6		292.5 ± 0.1
**11-Keto-5α-dihydrotestosterone**		214.3 ± 0.6		ND		ND
**11-Keto-5β-dihydrotestosterone**		214.0 ± 0.1		ND		ND
**11β-Hydroxy-5β-androstane-3,17-dione**		214.9 ± 0.1		217.4 ± 0.1		292.5 ± 0.1
**11β-Hydroxydihydrotestosterone**	406.306	214.9 ± 0.1	ND	ND	ND	ND
**11β-Hydroxy-5β-dihydrotestosterone**		213.1 ± 0.1		ND		ND
**11β-Hydroxyandrosterone**		213.0 ± 0.1		ND		ND

Table S6. $^{DT}CCS_{N2}$ and *m/z* values for all Girard's Reagent T derivatization major reaction products.

Compound	[MGT]$^+$ *m/z*	$^{DT}CCS_{N2}$ (Å^2)	[MGT$_2$]$^{+2}$ *m/z*	$^{DT}CCS_{N2}$ (Å^2)
**Adrenosterone**	414.275	216.7 ± 0.1	264.189	295.8 ± 1.1
**11β-Hydroxyandrostendione**	416.291	216.7 ± 0.1	265.197	295.7 ± 0.1
**11-Ketotestosterone**		216.7 ± 0.1		ND
**11-Keto-5β-androstanedione**		215.7 ± 0.1		297.7 ± 0.1
**11-Keto-5α-androstanedione**		216.7 ± 0.1		297.7 ± 0.1
**11β-Hydroxytestosterone**	418.306	217.6 ± 0.1	266.204	ND
**11β-Hydroxyandrostanedione**		216.7 ± 0.1		297.6 ± 0.1
**11-Keto-5α-dihydrotestosterone**		218.6 ± 0.1		ND
**11-Keto-5β-dihydrotestosterone**		218.6 ± 0.1		ND
**11β-Hydroxy-5β-androstane-3,17-dione**		215.7 ± 0.1		297.6 ± 0.1
**11β-Hydroxydihydrotestosterone**	420.322	218.9 ± 0.6	ND	ND
**11β-Hydroxy-5β-dihydrotestosterone**		217.6 ± 0.1		ND
**11β-Hydroxyandrosterone**		216.6 ± 0.1		ND

Table S7. $^{DT}CCS_{N2}$ and *m/z* values for all Girard's Reagent P derivatization major reaction products.

Compound	[MGP]$^+$ *m/z*	$^{DT}CCS_{N2}$ (Å^2)	[MGP$_2$]$^{+2}$ *m/z*	$^{DT}CCS_{N2}$ (Å^2)
**Adrenosterone**	434.244	220.4 ± 0.1	284.158	303.9 ± 0.1
**11β-Hydroxyandrostendione**	436.206	220.8 ± 0.1	285.166	303.9 ± 0.1
**11-Ketotestosterone**		220.8 ± 0.1		ND
**11-Keto-5β-androstanedione**		219.5 ± 0.1		303.9 ± 0.1
**11-Keto-5α-androstanedione**		220.8 ± 0.1		303.9 ± 0.1
**11β-Hydroxytestosterone**	438.276	220.8 ± 0.1	286.174	ND
**11β-Hydroxyandrostanedione**		220.8 ± 0.1		303.8 ± 0.1
**11-Keto-5α-dihydrotestosterone**		222.1 ± 0.1		ND
**11-Keto-5β-dihydrotestosterone**		222.1 ± 0.1		ND
**11β-Hydroxy-5β-androstane-3,17-dione**		219.5 ± 0.1		303.8 ± 0.1
**11β-Hydroxydihydrotestosterone**	440.291	222.1 ± 0.1	ND	ND
**11β-Hydroxy-5β-dihydrotestosterone**		221.2 ± 0.1		ND
**11β-Hydroxyandrosterone**		220.7 ± 0.1		ND

Table S8. $^{DT}CCS_{N2}$ and *m/z* values for all 2,4-dinitrophenylhydrazine (DNPH) derivatization major reaction products.

Compound	[MDNPH+H]+ *m/z*	$^{DT}CCS_{N2}$ (Å²)
**Adrenosterone**	481.208	226.3 ± 0.1
**11β-Hydroxyandrostendione**	483.224	227.3 ± 0.1
**11-Ketotestosterone**		227.3 ± 0.1
**11-Keto-5β-androstanedione**		226.3 ± 0.1
**11-Keto-5α-androstanedione**		226.6 ± 0.6
**11β-Hydroxytestosterone**	485.239	227.2 ± 0.1
**11β-Hydroxyandrostanedione**		227.2 ± 0.1
**11-Keto-5α-dihydrotestosterone**		227.2 ± 0.1
**11-Keto-5β-dihydrotestosterone**		227.2 ± 0.1
**11β-Hydroxy-5β-androstane-3,17-dione**		226.3 ± 0.1
**11β-Hydroxydihydrotestosterone**	487.255	228.2 ± 0.1
**11β-Hydroxy-5β-dihydrotestosterone**		227.2 ± 0.1
**11β-Hydroxyandrosterone**		226.3 ± 0.1

Table S9. $^{DT}CCS_{N2}$ and *m/z* values for all dansyl hydrazine (Dh) derivatization major reaction products.

Compound	[MDh+H]+ *m/z*	$^{DT}CCS_{N2}$ (Å²)	[MDh₂+H]+ *m/z*	$^{DT}CCS_{N2}$ (Å²)	[MDh₂+2H]+2 *m/z*	$^{DT}CCS_{N2}$ (Å²)
**Adrenosterone**	548.258	225.6 ± 0.1	795.336	271.8 ± 0.5	398.172	350.9 ± 4.2
**11β-Hydroxyandrostendione**	550.273	221.7 ± 0.1	797.351	274.6 ± 0.5	399.180	354.8 ± 0.1
**11-Ketotestosterone**		230.3 ± 0.1		ND		ND
**11-Keto-5β-androstanedione**		217.9 ± 0.1		273.3 ± 0.1		362.5 ± 0.1
**11-Keto-5α-androstanedione**		234.2 ± 0.1		273.3 ± 0.1		366.3 ± 0.1
**11β-Hydroxytestosterone**	552.289	229.3 ± 0.1	799.367	ND	400.187	ND
**11β-Hydroxyandrostanedione**		233.3 ± 0.1		274.3 ± 0.1		368.2 ± 0.1
**11-Keto-5α-dihydrotestosterone**		224.6 ± 0.1		ND		ND
**11-Keto-5β-dihydrotestosterone**		228.4 ± 0.1		ND		ND
**11β-Hydroxy-5β-androstane-3,17-dione**		218.8 ± 0.1		274.3 ± 0.1		364.4 ± 0.1
**11β-Hydroxydihydrotestosterone**	554.305	224.2 ± 0.1	ND	ND	ND	ND
**11β-Hydroxy-5β-dihydrotestosterone**		228.7 ± 0.6		ND		ND
**11β-Hydroxyandrosterone**		230.3 ± 0.1		ND		ND

Table S10. $^{DT}CCS_{N2}$ and *m/z* values for all 4-chlorophenylhydrazine derivatization major reaction products.

Compound	[MCl+H]+ *m/z*	$^{DT}CCS_{N2}$ (Å²)
**Adrenosterone**	425.199	213.0 ± 0.1
**11β-Hydroxyandrostendione**	427.215	213.0 ± 0.1
**11-Ketotestosterone**		213.0 ± 0.1
**11-Keto-5β-androstanedione**		213.0 ± 0.1
**11-Keto-5α-androstanedione**		214.4 ± 0.1
**11β-Hydroxytestosterone**	429.230	214.3 ± 0.1
**11β-Hydroxyandrostanedione**		214.3 ± 0.1
**11-Keto-5α-dihydrotestosterone**		214.3 ± 0.1
**11-Keto-5β-dihydrotestosterone**		213.9 ± 0.8
**11β-Hydroxy-5β-androstane-3,17-dione**		213.0 ± 0.1
**11β-Hydroxydihydrotestosterone**	431.250	214.7 ± 0.7
**11β-Hydroxy-5β-dihydrotestosterone**		214.3 ± 0.1
**11β-Hydroxyandrosterone**		213.4 ± 0.8

Table S11. $^{DT}CCS_{N2}$ and *m/z* values for all 4-bromophenylhydrazine derivatization major reaction products.

**Compound**	**[MBr+H]⁺ *m/z***	**$^{DT}CCS_{N2}$ (Å^2)**
**Adrenosterone**	469.148	215.9 ± 0.1
**11β-Hydroxyandrostendione**	471.164	216.9 ± 0.1
**11-Ketotestosterone**		216.9 ± 0.1
**11-Keto-5β-androstanedione**		216.2 ± 0.6
**11-Keto-5α-androstanedione**		217.2 ± 0.6
**11β-Hydroxytestosterone**	473.180	216.8 ± 0.1
**11β-Hydroxyandrostanedione**		217.8 ± 0.1
**11-Keto-5α-dihydrotestosterone**		217.8 ± 0.1
**11-Keto-5β-dihydrotestosterone**		216.8 ± 0.1
**11β-Hydroxy-5β-androstane-3,17-dione**		215.9 ± 0.1
**11β-Hydroxydihydrotestosterone**	475.195	218.1 ± 0.6
**11β-Hydroxy-5β-dihydrotestosterone**		217.8 ± 0.1
**11β-Hydroxyandrosterone**		216.8 ± 0.1

SUPPORTING FIGURES

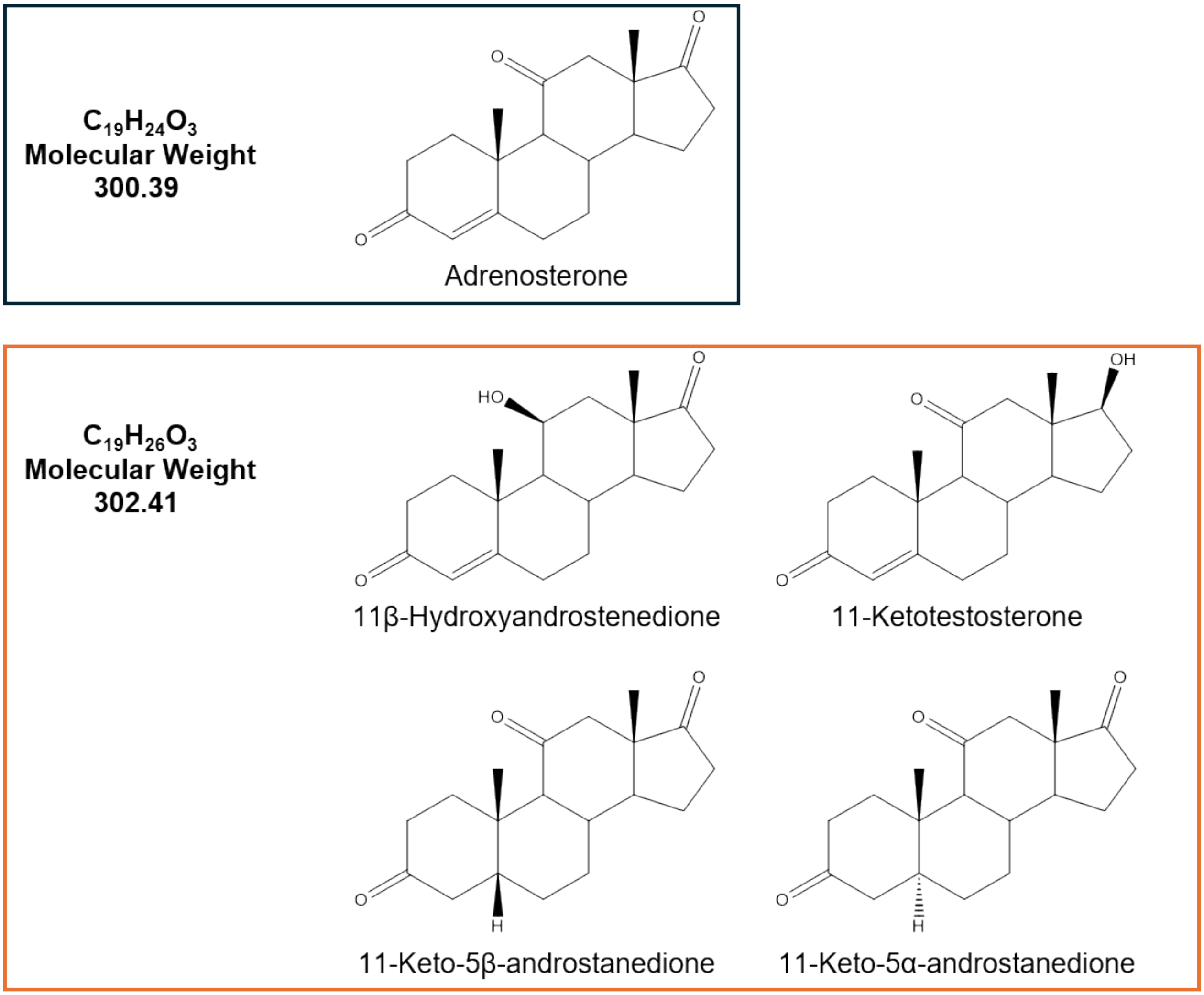

Figure S1. Chemical formula, molecular weight, and structure for the 11-oxosteroids surveyed in this work (grouped by isomers).

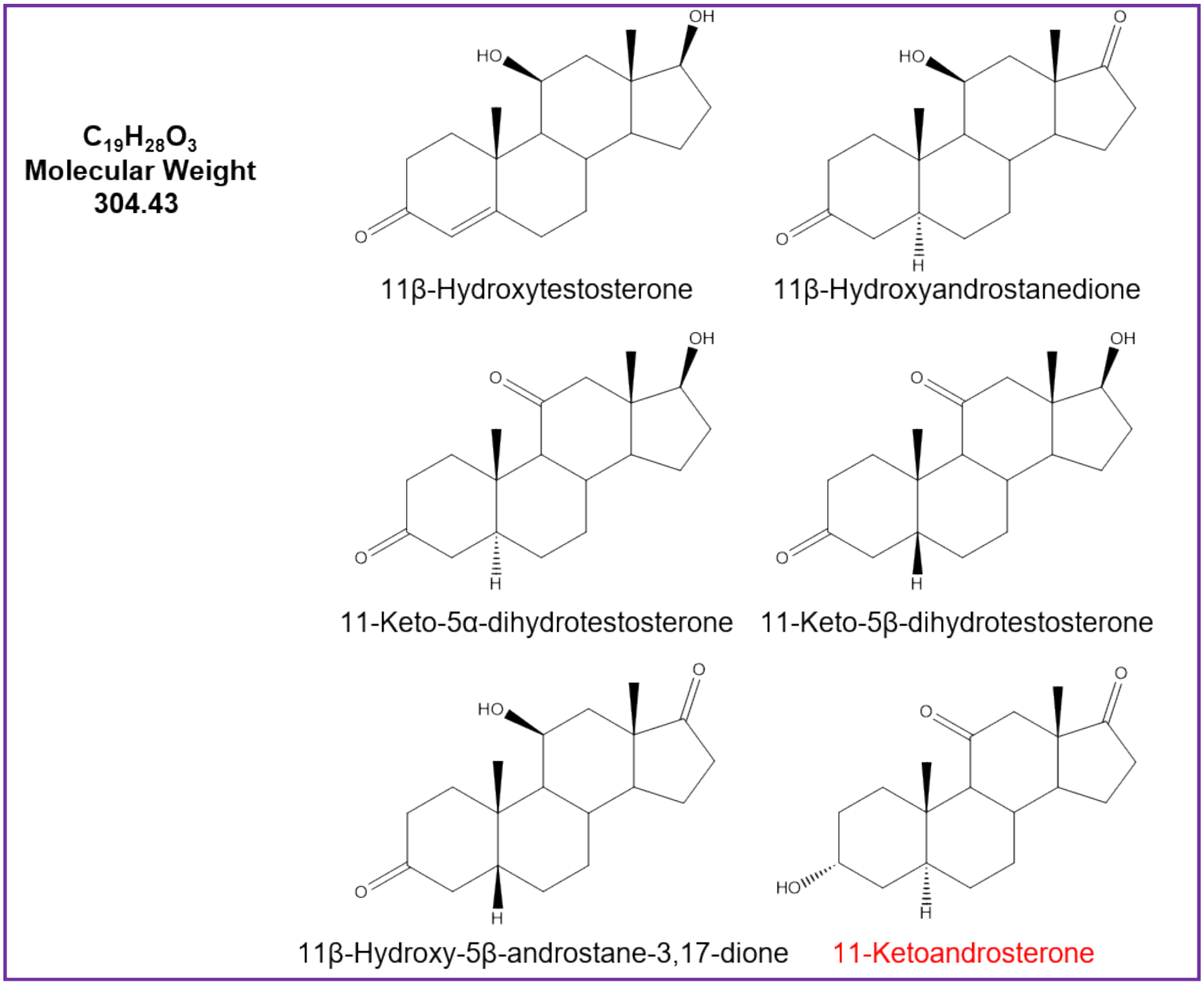

Figure S1. (continued) Chemical formula, molecular weight, and structure for the 11-oxosteroids surveyed in this work (grouped by isomers). *Note:* Compounds shown in red were not reactive with the derivatization reagents.

$C_{19}H_{30}O_3$
Molecular Weight
306.45

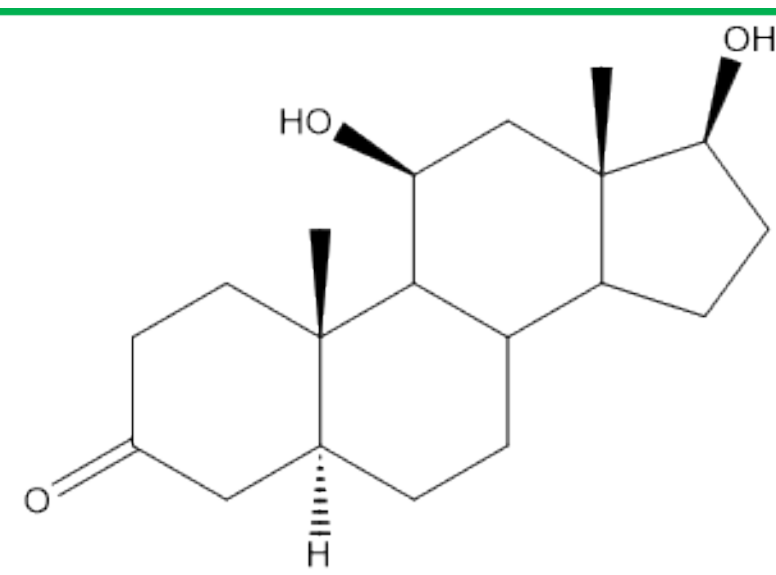

11β-Hydroxydihydrotestosterone

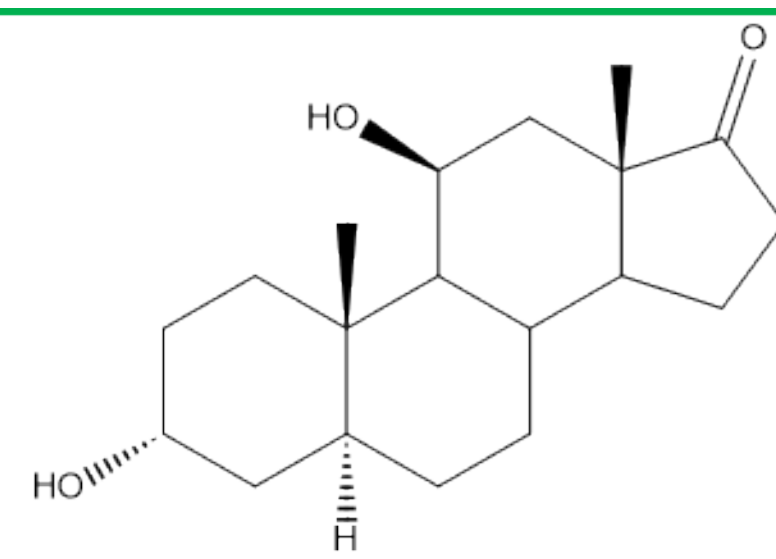

11β-Hydroxyandrosterone

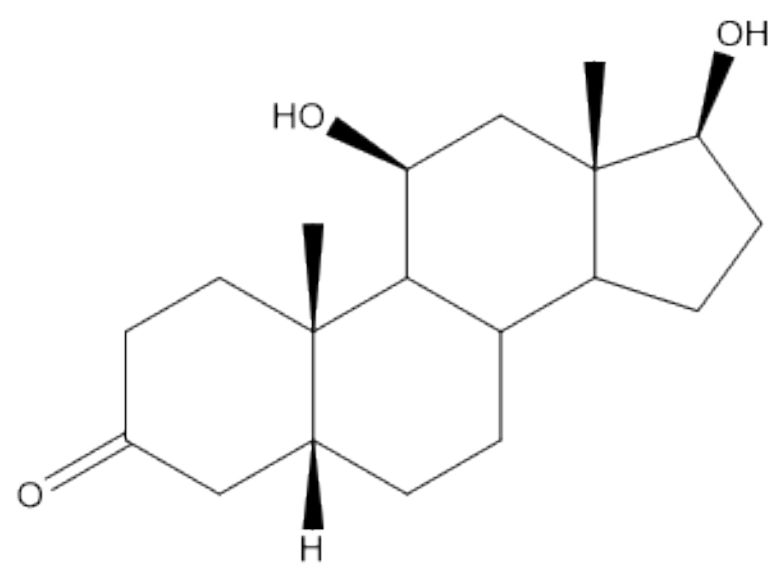

11β-Hydroxy-5β-dihydrotestosterone

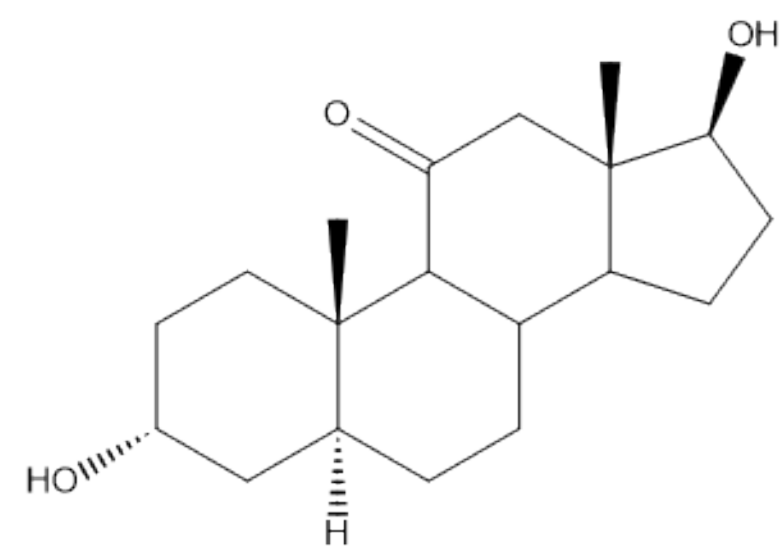

11-Keto-5α-androstanediol

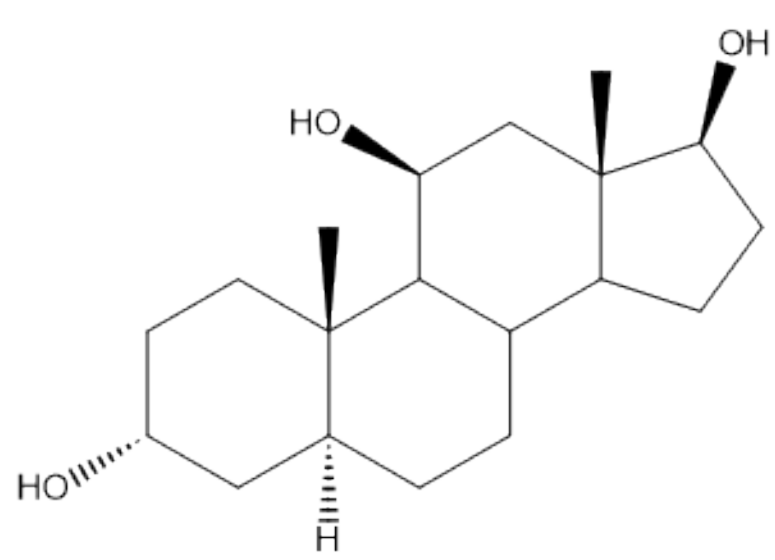

11β-Hydroxyandrostanediol

Figure S1. (continued) Chemical formula, molecular weight, and structure for the 11-oxosteroids surveyed in this work (grouped by isomers). *Note:* Compounds shown in red were not reactive with the derivatization reagents.

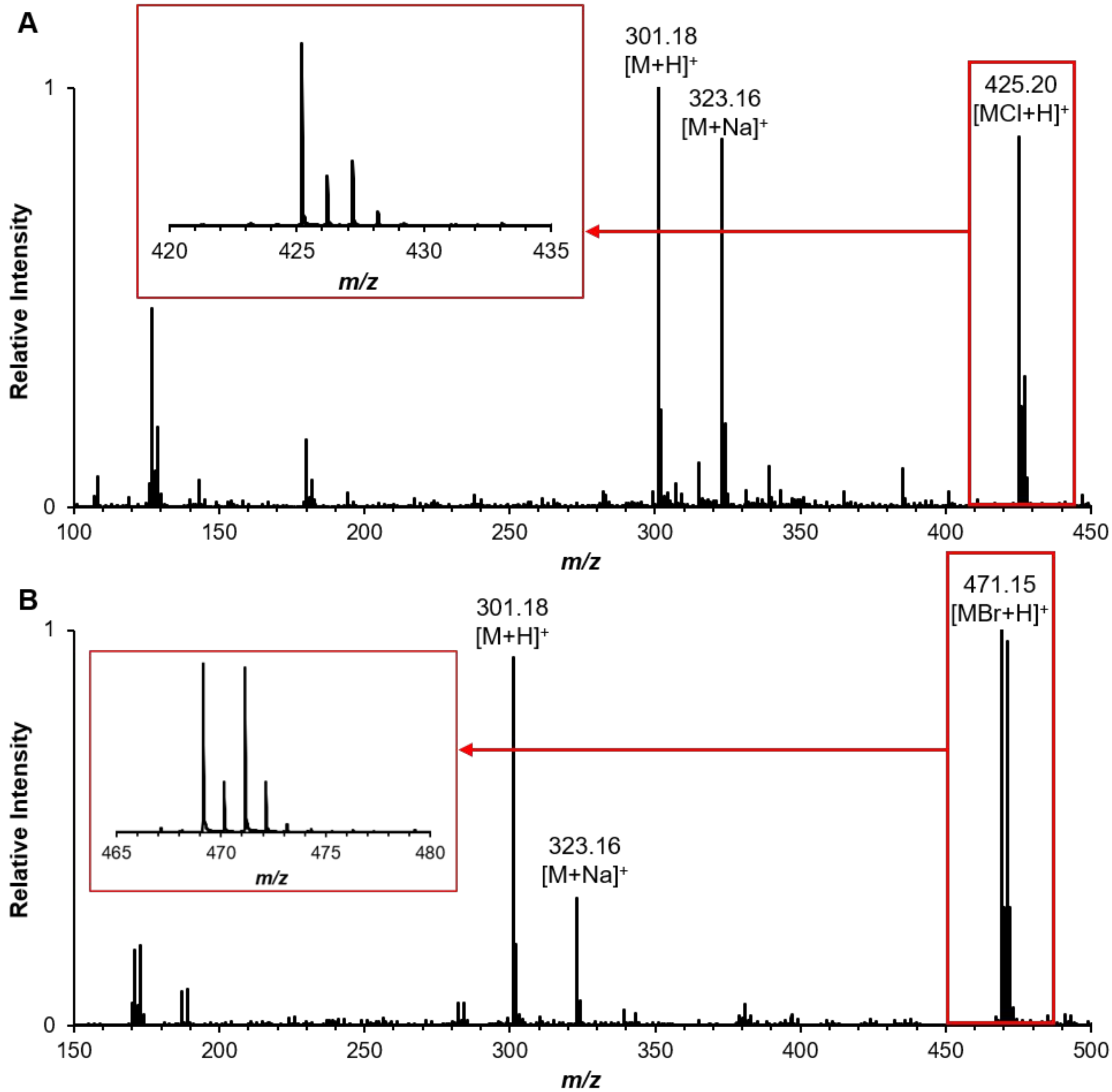

Figure S2. Mass spectra for adrenosterone derivatized with (A) 4-chlorophenylhydrazine, and (B) 4-bromophenylhydrazine. Inset zoom spectra show the characteristic isotopic patterns.
